# Supplementary material for: Patterns of Intron Gain and Loss in Fungi
Source: PLoS Biol. 2004 Nov 30;2(12):e422. doi: 10.1371/journal.pbio.0020422 (PMC532390; doi:10.1371/journal.pbio.0020422)
Supplement: Table S1 — Also available at http://genes.mit.edu/NielsenEtAl/. (4.3 MB ZIP). [file pbio.0020422.st001.zip › NielsenEtAl/html/968.html]

AN0229.1.NCU06986.1.MG01463.1.FG05653.1


```
 CLUSTAL W (1.82) Multiple Sequence Alignments - Introns Inserted


Sequence 1: NCU06986.1	902 aa
Sequence 2: MG01463.1	893 aa
Sequence 3: FG05653.1	897 aa
Sequence 4: AN0229.1	875 aa
Alignment Length: 937 aa
Number Identitical Residues: 282 aa
Alignment Score (without introns) 17811


MG01463.1 	----MS~SQED-------------QEKLTENKSLSG~LYSTLVPVLIASGAYLAIFLILR
NCU06986.1	MSDIPS~NTSD-------------TSEAKEPVSLSG1MIVTLVPILITSAIYLAIFLVLR
FG05653.1 	MGNFIS~WLEDNLPDPKSTKGNAGEARGPQPKSVSS1MVSTLVPVLVASAAYILIFLILR
AN0229.1  	----MA0DSEN---------------PQAASNSASS~LVTVLVPSLVVAGAMVLIFVILR
          	     :   .:                     * *. :  .*** *:.:.  : **::**

MG01463.1 	RSQRRWYAPRTYIGSLRPG2ERTPELPNGLFNWFGQFWKIPDTYALQHQGLDAYLYLRYM
NCU06986.1	KSNRRYYAPRTYLGSLREN~ERSPSLSNGLFSWVKDFWKIPDVYALQHQSLDAYLYIRYL
FG05653.1 	KSNRRFYAPRTYLGSLREH2ERSPALPGGWFGWIGTFWKIPDAYALQHQSLDAYLFIRFL
AN0229.1  	RSERRMYMPRTYLGVLRDS~QKTPPSSTGPLGWIKDMYKLPDEYVLQHHSMDAYLLLRFL
          	:*:** * ****:* **   :::*  . * :.*.  ::*:** *.***:.:**** :*::

MG01463.1 	RMSIVITFVGCCITWPILFPVNITGGGGQEQLDMLSYANVNAGSQEGRYRFFAHAITAWI
NCU06986.1	RMAVTICFVGCCITWPVLFPVNATGGNGLKQLDILTYGNINRETRYNRY--YAHVFISWI
FG05653.1 	RICCTICFVSLCITWPVLFPVNATGGNGKTELELLSYSNINIQSSKERNRLYAHCFIAWI
AN0229.1  	KIITMICFVGSCLTFPILFPVNATGGAGKQQLDILSFSNV---SETKYARFFAHALVAWV
          	::   * **. *:*:*:***** *** *  :*::*::.*:   :       :** : :*:

MG01463.1 	FYGFILYLIFRELVFYINLRQAFLLSPLYSRRISSRTVLFTSVPDAYLDEAHLRQVFGP-
NCU06986.1	FFGFVMYLIMRECIFYINLRQAFLISPLYSQRISSRTVLFTSVPEPYLDEQRLRKVFGA-
FG05653.1 	VYGFVMYTIMRECLFYVSVRQAFLLTPQYAKRISSRTVLFTSVPKDYLDEARIRTLFND-
AN0229.1  	FVAFVFFTVTRESLFYINLRQAYSLSRSYASRLSSRTVLFTTVPEEYQSVEKIRFMFGAN
          	. .*::: : ** :**:.:***: ::  *: *:********:**. * .  ::* :*. .

MG01463.1 	SVRNVWITYESKEVDELVKKRDERAYRLEKAEVKLIKLADKNRRAALKKGGSDAEADASK
NCU06986.1	SVKNVWITSETKEVDELVEERDKVAMRLEKAEVKLIKLANQIRRKAMSKGD---VNDVDK
FG05653.1 	SVKNVWIPGETKEVDEIIEERDEVAMKLEKGEVKLLKLCNKERIKSMKKSG----AEAEK
AN0229.1  	KVKNVWLTTDTAELAEKVNDRHAAAIKLEAAETKLIRMANAARLKALKKNRG-TGEQSTA
          	.*:***:. :: *: * ::.*.  * :** .*.**:::.:  *  ::.*. . :  :   

MG01463.1 	NEANQLDTESGSIAARWVPQGKRPTHRTGALGLIGSKVDSIDYCRDELHRLIPKTHEAQR
NCU06986.1	QAP--LDAESGSIAARWVPRNKRPTHRLGPLGLIGKKVDTIDWCREELTRLIPEAEAAQD
FG05653.1 	QNSGPTDPETGDLAARWIPQKKRPTHRTGPLGLIGKKVDTIEWGREELKTLIPKADEAQA
AN0229.1  	PEATEDDGESGSVAARWVRPKDRPTHRLKPI--IGKKVDTINWARSEIERLTPEIEALQT
          	  .   * *:*.:****:   .*****  .:  **.***:*:: *.*:  * *: .  * 

MG01463.1 	QYRSGTSKKIPGVFVEFRSQGEAEAAYQVVAHHRGLQMAPRYIGITPGDVIWSSLKVSWW
NCU06986.1	KYRDGAFKKVPGVFIEFRTQADAEGAAQILAHHRGLHMTPKYIGIRPNEIVWKSLAIPWW
FG05653.1 	NWLAGNYEKHSAVFVEFYTQSDAQAAFQTTTHHHALHMAPRHIGVKPDEVVWNSLKFPWW
AN0229.1  	QHRAGEAKLVSSVFVEFHTQADAQAAFQSVAHNLPLHMAPRYIGLEPTQIIWSNLRIKWW
          	:   *  :  ..**:** :*.:*:.* *  :*:  *:*:*::**: * :::*..* . **

MG01463.1 	QRVVRRYLVLAFIAALIIFWAFPVLVVGIITNIDKLKQDVSWLSWLNYIPD0AIMGVITG
NCU06986.1	QRVIRRYAVYAFITAMIIFWAIPVGVVGIISNVNYLKT-ISFLTWLDDIPG~FILGAVTG
FG05653.1 	QIVIRRYIIAAIIAILIIFWAIPVAIVGIIAQVNTIKT-LPGLTWIESIPS0VILGVISG
AN0229.1  	ERIIRYGASVAFVSAMVIFWAIPTAFVGSLSNINNLTDKVHFLRFINDVPD~WILGAITG
          	: ::*     *::: ::****:*. .** ::::: :. .:  * ::: :*.  *:*.::*

MG01463.1 	LLPSVALAILMSLVPIIMRL1CAKLSGEPSHSRCELFTQNAYFCFQIVQVFFVTT1VGSA
NCU06986.1	LLPSIALSILMSLVPIVIRI~CAKLSGEPSLSRVELFTQHAYFAFQVIQVFLVAT~VASS
FG05653.1 	LLPSVALSILMAMVPIFMRV~CAKQAGCVSISQAELYTQNTYFVFLVLQVFLVQT~LANS
AN0229.1  	LLPTILMAVLMALVPIVLRL1MAKIGGAPNLAAVELSTQNFYFFFQVVQVFLVVT~LSSA
          	***:: :::**::***.:*:  ** .*  . :  ** **: ** * ::***:* * :..:

MG01463.1 	GTELGPQLQKMALDPQLIPQTLADTLPKPGNFYNNYFLVQGLTIAASVLSQIVGFVIFQL
NCU06986.1	ATAVA---KQIADNPGSVTKLLSENLPKSSNFYISYFIVQGLSIATSVLTQVVGFFVFNL
FG05653.1 	FVSSI---VTIVQDPSQVFTMLSSSIPTASNFYISYFIVQGLGIATSVLTQVVGCVIFNL
AN0229.1  	ATSVV---TKIVSNPTSATSLLANNLPLSANFYISYIVLQGLSFSSGALLQISGLILGKI
          	 .        :. :*      *:..:* ..*** .*:::*** :::..* *: * .: ::

MG01463.1 	MYRFLASTPRALYQKWATLSAISWGSTLPVYTMITCIA~IIYAVITPLVLGFACLAMCLF
NCU06986.1	VYKFLANTPRALYTKWANLSAISWGSTMPVYTNIVVIA1IAYATIAPLMLGWATVAMGLF
FG05653.1 	LYKFLASTPRAMYNKWTTLSALTWGSLMPVYTNIAVIS1IVYAVIAPLMLFWSTLGMALF
AN0229.1  	LGRLLDNTPRKMYSRWSNLAGLGWGTVYPVFTLLAVIA1ITYSCIAPLVLGFATIGLYLF
          	: ::* .*** :* :*:.*:.: **:  **:* :. *: * *: *:**:* :: :.: **

MG01463.1 	YIAWRYNVLFVTDTKIDTRGLIYPRALKQLFVGVYVAEICMIGLFGAST-----AIGPAV
NCU06986.1	YLAWRYNVLFVTDTQIDTRGLIYPRAIKQLYTGIYLAEICMIGLFGASV-----APGPLV
FG05653.1 	YLAYRYNILFVTETKIDTRGLIYPRALKQLFVGVYLAEVCLIGMFIVSK-----AAGPAA
AN0229.1  	YFAYRYNLLYVSNADIDTQGKTFARALQQILVGCYLLVVCLIGLFAIGTGTDPIALGPMI
          	*:*:***:*:*:::.***:*  :.**::*: .* *:  :*:**:*  . .:.. * **  

MG01463.1 	LMAA1LLLFTVLFQLTLTNALDPLLYNLPQTLLAEEELLLLAP-------QNGALSAGPS
NCU06986.1	LMVI~FLIFTILFHISMNAALNPLLYNLPLTLLAEEEGPLLDTPVDSHTAENGLGGEPRN
FG05653.1 	LMVI~FLIFSILFHMTMAKALNPLLYNLPRSLEVEEERIQQSAQ--GSELEDGQVLNN-N
AN0229.1  	LTII~LLVTMIIYHVSLNAALDPLINYLPRNLEAEEEALLSQET-------NGAKDEYDG
          	*    :*:  :::::::  **:**:  ** .* .***              :*      .

MG01463.1 	GATGTT-------MATNGFASNDAHSPADIKDTEAAAIAT--VP---SAKKGNILSRFFM
NCU06986.1	GVSPTTGANYTEKVPSNGTNGAQSRAEDPILGAKEALDASRHQPNHVTGKKPNFLVKFLK
FG05653.1 	GVTNAT---------TNGVENGDAKSTRVGKFVPGGVGGVQ--------KKGNFFSKWLK
AN0229.1  	SASPSA---------------ADGQTADNVDSAEKGLTQAN-----PAEPKTNFLTKYLR
          	..: ::                :.::      .  .           :  * *:: ::: 

MG01463.1 	PWMYADYATLRKLVPQGTELDLDLDNLYSDEVNGNAYYPPAVTSTPPLLWIPRDA--AGV
NCU06986.1	PWIYSDYDTLRAYLPRGVQLDP-----YTEEVERDAYCPPNVTSDVPLLWIPSDANGAGV
FG05653.1 	PWIYADYATMRQLVPHESTMGLD----YTEQVERDAYFPPSITSETPILWIPADP--AGI
AN0229.1  	PDKYSGYRQLRTLVPNGTETVS-----YPPEVERNAYYHPAISAQAPLLWIPRDE--MGI
          	*  *:.*  :*  :*.           *. :*: :**  * :::  *:**** *    *:

MG01463.1 	SKQEIAHTGKVIHITDEGCELDENGKLVWDRESTRPPVWEEKVPY
NCU06986.1	SRQEVEHTSRVVPITDEGCELDEKGHLVWDREGTRPPIWEEKIYY
FG05653.1 	SKQEVLHTSKVIPISDEGCTLNDKNNIEWDTEGARPPIWTEKIYY
AN0229.1  	SAQEVAHTSRVIPITDEDAWLDEKNHIVWNVEKGVPPIFEEKIYY
          	* **: **.:*: *:**.. *:::.:: *: *   **:: **: *
```
